# Identification of an *RSPH4A* Founder Variant and Newborn Screening for Primary Ciliary Dyskinesia

**DOI:** 10.1001/jamanetworkopen.2025.30551

**Published:** 2025-09-05

**Authors:** Wilfredo De Jesús-Rojas, Katherine P. Guerra-Torres, Alondra J. Rosado-Ayala, Edwin L. Acevedo-Soto, Marcos J. Ramos-Benitez, Ricardo A. Mosquera

**Affiliations:** 1 Department of Basic Sciences, Ponce Health Sciences University, Ponce, Puerto Rico; 2 Department of Pediatrics, McGovern Medical School, University of Texas Health Science Center at Houston, Houston

## Abstract

This cross-sectional study estimates the prevalence of an *RSPH4A *variant identified in Puerto Rico in an effort to evaluate the feasibility of targeted newborn screening for primary ciliary dyskinesia (PCD).

## Introduction

Primary ciliary dyskinesia (PCD) is a rare genetic disorder caused by dysfunctional motile cilia and presents with unexplained respiratory distress in approximately 80% of full-term neonates; early diagnosis is critical to mitigate long-term complications from chronic airway inflammation and infection.^1,2^ Diagnosis is complicated by marked genetic heterogeneity (>55 genes implicated), with disease-causing variants often varying by ancestry.^3^ In Puerto Rico, an *RSPH4A* variant (c.921 + 3_921 + 6delAAGT) has been reported, accounting for the 66.7% of all pathogenic alleles and suggesting a strong founder effect.^4,5^ However, its prevalence in the general newborn population remains unknown, limiting efforts to estimate disease burden and evaluate the feasibility of targeted newborn screening.

## Methods

We conducted a deidentified, population-based, cross-sectional study to estimate the prevalence of the *RSPH4A* variant using dried blood spots from newborns screened through the US Department of Health. From June to July 2024, samples were randomly selected across Puerto Rico, representing approximately 12% of annual births. DNA was extracted and analyzed by targeted polymerase chain reaction using fluorescein amidite–labeled primers and capillary electrophoresis on a sequencing instrument (ABI 3500 Genetic Analyzer; Applied Biosystems) to identify wild-type, heterozygous, and homozygous genotypes. Allele frequency was calculated to estimate the population-level and birth prevalence of PCD based on Hardy-Weinberg equilibrium. This study was approved by the institutional review board of Ponce Health Sciences University and adheres to STROBE reporting guidelines. In accordance with the Common Rule 45 CFR §46, written informed consent from parents or legal guardians was not required as samples were obtained from residual, previous collected specimens and fully deidentified. Statistical significance was determined by the χ^2^ test using GraphPad Prism version 10.3.1 statistical software and was set at 2-sided *P* < .05.

## Results

Among 1994 newborns, 25 (1.3%; 95% CI, 0.8%-1.8%) were heterozygous carriers and 1 (0.05%; 95% CI, 0.001%-0.3%) was homozygous for the *RSPH4A* variant (Table). The remaining 1968 (98.7%; 95% CI, 98.1%-99.1%) had wild-type *RSPH4A*. The estimated allele frequency was 2.2% (95% CI, 1.7%-2.2%) by Hardy-Weinberg equilibrium and 0.7% (95% CI, 0.5%-1.0%) by direct calculation. A χ^2^ test showed a significant deviation from Hardy-Weinberg equilibrium (χ^2^ = 46.62; *P* < .001), consistent with a strong founder effect. The projected prevalence of PCD due to this variant is 0.05%, or 502 cases per million (95% CI, 344-659 cases per million), corresponding to approximately 1624 individuals affected in Puerto Rico (95% CI, 263.2-9126.4 individuals). Based on the 16 360 live births reported in Puerto Rico in 2024, an estimated 8 newborns with PCD due to the *RSPH4A *founder variant are expected annually (95% CI, 1.3-45.9 newborns per year).

**Table.** Genotype Frequencies and Projected Prevalence of the *RSPH4A* Variant in Puerto Rican Newborns

Genotype	Newborns, No. (%) [95% CI] (N = 1994)	Interpretation
Wild-type	1968 (98.7) [98.1-99.1]	Proportion of individuals without the *RSPH4A* variant
Heterozygous carrier	25 (1.3) [0.8-1.8]	Proportion of individuals carrying 1 copy of the *RSPH4A* variant
Homozygous	1 (0.05) [0.001-0.3]	Estimated proportion of individuals who are homozygous for the *RSPH4A* variant
Allele frequency (Hardy-Weinberg–based estimation), % (95% CI)	2.2 (0.4-5.3)	Estimated frequency of the variant allele using Hardy-Weinberg assumptions
Allele frequency (direct calculation), % (95% CI)	0.7 (0.5-1)	Observed frequency of the variant allele directly from genotype counts
Estimated cases, No./1 000 000 population (95% CI)	502 [344-659]	Estimated No. of homozygous individuals per 1 million based on projected prevalence
Estimated individuals with PCD in Puerto Rico, No. (95% CI)	1624 (263.2-9126.4)	Extrapolated No. of affected individuals in the island’s total population
Estimated annual births with PCD, No. (95% CI)	8 (1.3-45.9)	Expected No. of newborns with PCD each year in Puerto Rico

Abbreviation: PCD, primary ciliary dyskinesia.

## Discussion

To our knowledge, this cross-sectional study is the first population-level estimate of the *RSPH4A* founder variant using newborn screening data. The high carrier frequency and marked divergence from global reference datasets confirm a strong founder effect. According to gnomAD v4 Genomes, the global allele frequency of the *RSPH4A* variant is 0.03%, with a higher frequency of 0.2% among individuals of Latino and/or admixed American ancestry.^6^ In comparison, our observed frequency in Puerto Rican newborns (2.2%) is approximately 80 times higher than the global estimate and 9 times higher than the Latino and/or Admixed American group. These findings suggest that PCD due to this variant remains underdiagnosed in Puerto Rico, where over 1600 individuals may be affected. Early diagnosis is essential to slow disease progression through airway clearance, infection control, and timely referral. Integrating targeted screening for this founder variant into the existing newborn screening framework could help close this diagnostic gap, particularly in genetically isolated or founder-effect populations. The polymerase chain reaction and fragment analysis method used in this study offers a feasible, variant-specific approach that could serve as a pilot model. While we focused on 1 intronic deletion, the screening panel could be expanded to include other PCD-related genes relevant to specific populations. Limitations include the focus on a single variant and reliance on Hardy-Weinberg assumptions. Our estimates may miss individuals with other pathogenic or compound heterozygous genotypes. Further research is needed to assess clinical outcomes and implementation feasibility. In Puerto Rico, where founder variants drive many rare diseases, genomic data can inform precision public health. Broader access to diagnostics, specialized care, and counseling is key to improving PCD outcomes.

## Notes

Supplement.
Data Sharing Statement
