# Supplementary material for: Identification of an RSPH4A Founder Variant and Newborn Screening for Primary Ciliary Dyskinesia
Source: JAMA Netw Open. 2025 Sep 5;8(9):e2530551. doi: 10.1001/jamanetworkopen.2025.30551 (PMC12413642; doi:10.1001/jamanetworkopen.2025.30551)
Supplement: Supplement. — Data Sharing Statement [file jamanetwopen-e2530551-s001.pdf]

Data Sharing Statement

De Jesús-Rojas. Identification of an RSPH4A Founder Variant and Newborn Screening for Primary Ciliary Dyskinesia. *JAMA Netw Open*. Published September 05, 2025. doi:10.1001/jamanetworkopen.2025.30551

Data
Data available: Yes
Data types: Deidentified participant data
How to access data: wdejesus@psm.edu
When available: With publication

Supporting Documents
Document types: Other (please specify)
Additional Information: IRB
How to access documents: wdejesus@psm.edu
When available: With publication

Additional Information
Who can access the data: researchers whose proposed use of the data has been approved
Types of analyses: or a specified purpose
Mechanisms of data availability: after approval of a proposal,
Any additional restrictions: None
